# Dr. T. Daureeawoo

**Published:** 1983

**Authors:** 


### Miscellanea

### DR. T. DAUREEAWOO

It is with great pleasure that one announces that Dr. Tayab Daureeawoo has been elected Fellow of the Royal College of Physicians of Ireland in 1982. Dr. Daureeawoo qualified at Bristol University in 1964. He was appointed a House Surgeon at Frenchay Hospital and a House Physician at Ham Green Hospital. He returned to Mauritius in 1965 to work in the public service and had a hospital appointment. He was made a medical officer at the hospital of Montague Longue for 3 years.

In 1971 he took the examination for the M.R.C.P. in Dublin, and he joined Professor A. Goldberg, F.R.S. Edinburgh, in the Glasgow Western Infirmary. He returned to Mauritius and has continued both a busy hospital and private practice.

We in Bristol send our congratulations to Dr. Tayab Daureeawoo, who has helped to raise the standards of medicine in Mauritius. He has continued to support Bristol University. He has visited Bristol regularly, and any graduate of the University who is lucky enough to visit Mauritius will be assured of a wonderful and generous welcome from the Daureeawoo family. Dr. Daureeawoo married when he was in Bristol, and has a very happy family with four children. His eldest son Isaac is in the final year of his medical course in Bristol University, and he will bring further distinction to the family and to the medical fraternity of Mauritius.

Dr. Tayab Daureeawoo has brought great distinction to Mauritius and to Bristol University. The University and especially his friends send greetings to the family and to the physicians and surgeons of the island of Mauritius, who have largely based their medical practice on that of the British Isles. We rejoice in the honour conferred on one of those who has maintained the highest standards of medical practice, and has continued to support Bristol University ever since he qualified in 1963.

